# Behavioral measures and factors influencing active use of health services in school hospitals by university students

**DOI:** 10.3389/fpubh.2025.1723284

**Published:** 2025-12-12

**Authors:** Jiarui Sun, Xue Zhou, Xuemei Li, Jiayi Liu, Shuke Chen, Yujuan Yang, Hainan Song

**Affiliations:** 1 School of Public Health, Mudanjiang Medical University, Mudanjiang, China; 2 School of Health Management, Mudanjiang Medical University, Mudanjiang, China; 3 School of Medical Imaging, Mudanjiang Medical University, Mudanjiang, China

**Keywords:** Delphi method, health service, university, student, behavioral measures

## Abstract

**Objective:**

To evaluate the level and factors associated with university students’ active utilization of health services in school hospitals across four universities in a Chinese province, and to put forward relevant strategies for improving their health literacy and active service use.

**Methods:**

A four-dimensional, 16-item scale was developed via the Delphi method to assess students’ active use of school hospital health services. Multiple regression was applied to explore factors associated with such utilization.

**Results:**

The overall average score for university students’ active utilization of health services in school hospitals was (60.68 ± 10.82). Among the four dimensions, university students had the highest score in communication and dialogue behavior (15.64 ± 3.05) and the lowest score in accessible behavior (14.55 ± 3.03). Self-assessed physical status and only-child status were statistically significantly associated with students’ active use of school hospital health services.

**Conclusion:**

The scale exhibits good reliability and validity, which provides a good basis for measuring university students’ active use of health services. Strategies to support students’ active use of health services may include enhancing school hospital service accessibility, strengthening service-related publicity and education, and improving students’ health initiative.

## 1 Introduction

The “Healthy China 2030” strategy envisions a comprehensive institutional system for promoting the health of all people, greater coordination in health sector development, widespread adoption of healthy lifestyles, continuous improvement in the quality of health services and health security, a thriving health industry, and the achievement of health equity. It also sets the goal of elevating China’s main health indicators to reach the levels of high-income countries.

As a key component of high-quality population development under the Healthy China initiative, university students represent an important reserve of future talent. School hospitals, as the most accessible and convenient primary healthcare institutions within universities, serve as the first line of defense in safeguarding student health. The National Development and Reform Commission of the People’s Republic of China issued a notice in 2024 entitled “Further Improving the Quality of Medical Security for University Students,” which proposes to enhance the level of medical services and consolidate efforts to improve medical security for university students by strengthening the construction and management of on-site medical and healthcare institutions. This directive establishes a policy anchor for comprehensively improving the service quality of school hospitals and effectively improving the health security of university students receiving care in school hospitals. For young university students, the integration of sports, health, and education is crucial to relevant to fostering proactive health practices and supporting their healthy development (1). Improving university students’ health literacy involves both policy guarantees and the promotion of proactive health behaviors, which aligns with the United Nations Sustainable Development Goal (SDG) 3.4 proposed by the World Health Organization: *“Noncommunicable diseases and mental health: By 2030, reduce by one third premature mortality from noncommunicable diseases through prevention and treatment and promote mental health and well-being.”* Such efforts are associated with providing intellectual support and talent for advancing population health and well-being. University students have developed proactive health behaviors related to the active acquisition of health information (2) and the adoption of healthy lifestyles (3). However, studies focusing on factors associated with university students’ active utilization of health services in school hospitals are scarce. This study aims to assess the level of such active utilization, explore related influencing factors, and put forward relevant strategies for supporting university students’ active use of health services and improving their health literacy.

## 2 Materials and methods

This paper employs a mixed- approach, combining qualitative and quantitative methods. The qualitative component uses the Delphi method for expert consultation, conducting two rounds of consultation to determine the measurement indicators of the level of college students’ proactive use of school hospital health services. This process resulted in a high-quality measurement scale developed through expert consensus. The quantitative component involved a questionnaire survey to collect data from college students on their proactive use of school hospital health services, followed by statistical analyses—such as multiple regression—to investigate the factors influencing these behaviors. The combination of these two methods ensures the professional validity of the measurement tool and strengthens the objectivity and accuracy of the findings, providing theoretical support and data-driven decision-making to inform improvement strategies.

### 2.1 Data sources

This study involved two stages: the scale development stage and the empirical analysis stage.

The data used for the development stage of the scale was derived from a behavioral index measurement based on the DART (Dialogue-Access-Risk Assessment-Transparency) Value Co-Creation Model, conducted by the research team from January to March 2025. Experts from universities, health administrative departments, and primary healthcare institutions, most of whom possess over five years of relevant work experience in health policy, primary healthcare, and health management, participated in this process.

The data used in the empirical analysis phase originates from a questionnaire survey conducted by the research team between April and June 2025. The team focused on a Hei Longjiang province in China, randomly selecting four universities as sample sources. Participants in the survey included university students from various disciplines who had been enrolled for over six months.

### 2.2 Methods

#### 2.2.1 Delphi method

The Delphi method is a research technique whereby experts engage in multiple rounds of surveys to achieve consensus (4). This method is primarily characterized by anonymity, allowing experts to modify their opinions based on aggregated feedback, thereby facilitating a gradual convergence of views within the expert group and culminating in a more coherent conclusion (5). The Delphi method is widely recognized within various domains of the medical and health fields, including occupational health (6), healthcare (7), and mental health (8). In this study, the scale development phase employed the Delphi method to conduct two rounds of expert consultations, determining the measurement indices for the behavior of university students toward active use of health services in school hospitals. This approach effectively fosters the exchange of opinions among experts, mitigating decision-making biases and reaching a high-quality consensus (9).

#### 2.2.2 Statistical analysis

SPSS 26.0 statistical software was used for statistical analysis. Quantitative data were described as mean ( $\bar{x}$ ) ± standard deviation (𝑠), with independent sample t-tests and analysis of variance used for group comparisons. Multiple regression analysis was used to examine the influencing factors of university students’ active use of health services in school hospitals. The threshold for statistical significance was set at *p* < 0.05.

## 3 Results

### 3.1 Scale development stage

#### 3.1.1 Construction of measurement indicators for proactive use of health services in school hospitals

Through literature review, the DART value co-creation model was integrated into the primary health field (10), adapting its four core dimensions—dialogue, access, risk assessment, and transparency—into communication dialogue, accessibility, risk assessment, and transparency. A pool of items composed of four first-level indicators and 16 s-level indicators was initially constructed.

#### 3.1.2 Determination of behavior measurement indicators of active use of health services in school hospitals based on the Delphi method

The first round of consultation involved 15 experts, while the second round included 12 experts. Each round issued 15 and 12 consultation forms, respectively, with a 100% effective recovery rate for all questionnaires. In the first round, three experts proposed detailed revisions to the measurement indicators, resulting in the replacement of the “risk assessment” dimension in the first-level indicator with the “process trust” dimension and the substitution of “effective communication” in the second-level indicator under “communication and dialogue” with “communication channels,” indicating high expert engagement (11). The expert authority coefficients were 0.84 and 0.83, both exceeding 0.7, signifying a high level of expert authority and reliability of the consultation results. The Kendall coefficient of harmony (W) was employed to assess the consistency of expert opinions. The coordination coefficients of the two rounds of expert consultation ranged from 0 to 1, with observed statistically significant differences (*p* < 0.05). The coordination coefficients exhibited stability, suggesting consensus among the experts (Table 1).

**Table 1:** Coordination coefficient of expert opinions.

Measurement metrics	First round			Second round		
	W	c2	*P*	W	c2	*P*
First-level indicators	0.218	9.81	0.034	0.375	13.86	0.003
Second-level indicators	0.302	70.26	0.032	0.454	72.33	0.001
			*P* < 0.05			*P* < 0.05

The Likert 5-point scoring method was used to evaluate the indicators, ranging from 1 to 5 points based on importance. In the first round of consultation, the average item importance score was 3.82, with a coefficient of variation from 0.07 to 0.36. Retention criteria included an average importance score exceeding 3.50, a perfect score ratio greater than 20.00%, and a coefficient of variation below 0.25 (8). Subsequently, four indicators under one first-level indicator and another second-level index were replaced based on the item retention criteria and the opinions of the expert group, resulting in a final set of 16 indicators for the second round. The average importance score for all indicators in the second round exceeded 3.50, with a perfect score ratio greater than 20.00% and a coefficient of variation below 0.25. The specific measurement indicators and scoring criteria are summarized in Table 2.

**Table 2:** Results of the second round of expert consultation.

Dimensions of measurement	Measurement metric description	Mean	Standard deviation	Coefficient of variation	Full score ratio
Dialogue	Communication channels	4.42	0.51	0.12	33.33
	Emotional support	4.67	0.49	0.11	53.33
	Individual case diagnosis and treatment	4.67	0.49	0.11	53.33
	Decision-making participation	4.67	0.49	0.11	53.33
Access	Psychological identification	4.92	0.29	0.06	73.33
	Medical service convenience	4.67	0.49	0.11	53.33
	Health promotion and education	4.83	0.39	0.08	66.67
	Economic burden	4.67	0.49	0.11	53.33
Risk assessment	Professional trust	4.17	0.72	0.17	26.67
	Relational trust	4.33	0.65	0.15	33.33
	Effectiveness trust	4.42	0.51	0.12	33.33
	Privacy trust	4.33	0.65	0.15	33.33
Transparency	Process transparency	5.00	0.00	0.00	100.00
	Fee transparency	4.75	0.45	0.10	60.00
	Disease comprehension	4.67	0.49	0.11	53.33
	Medical record accessibility	4.75	0.45	0.10	60.00

#### 3.1.3 Reliability and validity test of the scale

Before the formal survey, a small-scale pre-survey was conducted on the measurement scale resulting from expert consultations to evaluate its quality and the feasibility of empirical analysis. In this study, both Cronbach’s *α* coefficient and exploratory factor analysis were used to test the reliability and validity of the scale (12). Data analysis showed good reliability and validity of the scale. The overall Cronbach’s α coefficients was 0.95 (>0.7), with dimension-specific coefficients at 0.884, 0.837, 0.906, and 0.917, confirming the reliability of the scale was good. Additionally, the KMO (Kaiser-Meyer-Olkin) was 0.925 (>0.5), and the significance level of Bartlett’s test of sphericity was less than 0.001, indicating appropriateness for factor analysis. Finally, the correlation coefficients between the factors of the measurement scale and the total scale was greater than the correlation coefficient between the factors, indicating that the scale had good validity. Hence, this scale can serve as a foundational tool for assessing university students’ active use of health services.

### 3.2 Empirical analysis stage

#### 3.2.1 Survey tools and basic information of the study participants

The behavior measurement scale for university students’ active utilization of school hospital health services, derived from the Delphi method, was used to evaluate their level of use. Students scored the scale items according to their actual behavior and perceptions. The scale contains a total of 16 questions across four dimensions, employing the Likert 5-point scoring method with the following assignments: strongly disagree = 1, disagree = 2, neutral = 3, agree = 4, and strongly agree = 5. The research team distributed a total of 750 questionnaires, collected all 750 responses, and eliminated those with missing key variables and not completed in carefully (all respondents chose the same option for all questions), ultimately retaining 649 valid questionnaires, resulting in an effective response rate of 86.53%. Among the 649 surveyed university students, the average age was 20.12 ± 1.48 years. The distribution of respondents from urban and rural household was approximately equal, with a male-to-female ratio of about 1:3. Most respondents’ families reside in urban areas, while freshmen constituted the largest proportion of participants, followed by juniors, sophomores, seniors, and others. The majority of the surveyed university students recounted experiences of being ill and seeking treatment; more than half reported monthly living expenses below 1,800 yuan.

#### 3.2.2 Level of active use of health services in school hospitals

This study describes the score using the mean ± standard deviation, with higher scores indicating higher levels of active use of health services among university students. The scoring criteria categorize scores as follows: <8, 8–12, 12–16, and >16 points, indicating worse, general deviation, general preference, and good, respectively. The overall average score across all dimensions is derived from the aggregated scores of university students’ active use behavior, categorized as poor (<36), average (36–64), and good (>64) (Table 3).

**Table 3:** Scoring of university students’ proactive use of campus health services.

Dimension	Score	Item Average
	(Mean ± Standard deviation)	(Mean ± Standard deviation)
Dialogue	15.64 ± 3.05	3.91 ± 0.76
Access	14.55 ± 3.03	3.64 ± 0.76
Risk assessment	15.07 ± 2.98	3.77 ± 0.74
Transparency	15.43 ± 2.94	3.86 ± 0.73
Total score	60.68 ± 10.82	

The overall average score for active use of school hospital health services was 60.68 ± 10.82 points. Based on the score evaluation criteria, the overall level of active use of these services was deemed average.

Of the four dimensions, university students achieved the highest scores in communication and dialogue behavior (15.64 ± 3.05). This may be attributed to students in a higher education context having better communication skills and cognitive abilities (13), allowing them to articulate their health concerns clearly and comprehend their healthcare providers’ medication guidance. As a result, the quality of communication and mutual satisfaction between students and healthcare providers are improved to a certain extent. The unique open and inclusive humanistic environment found on university campuses promotes equitable dialogue between students and doctors, enhancing communication effectiveness and fostering positive feedback. Students are more willing to communicate with doctors, and doctors who are more willing to answer questions that arise during medical treatment. Regarding communication channels, in addition to traditional face-to-face communication between doctors and patients, the campus environment provides students with more diversified feedback channels, which also makes communication and dialogue smoother to a certain extent.

The average transparency behavior score of university students was 15.43 ± 2.94, reflecting a general preference. Information regarding the medical treatment process and charging standards of school hospitals is usually public. Since 2017, the implementation of the “zero mark-up policy on pharmaceuticals” in school hospitals across China has resulted in the prices of conventional drugs in school hospitals generally lower than those in off-campus pharmacies and hospitals, which provides university students with reasonable expectations regarding medical expenses and minimizes the burden of medical expenditure. Through school-led health education, students gain a clear understanding of the diagnosis, treatment processes, and service scope of their respective school hospital, fostering a sense of psychological security.

The process trust behavior score for university students was 15.07 ± 2.98, indicating general preference. This score reflects the trust of university students in the professional ability and privacy protection measures implemented by school hospitals. While students may trust the professionalism of the doctors in the school hospital, the functional role of these facilities is often limited to addressing common and frequent diseases among students (14). Consequently, the ability of school hospitals to manage complex health conditions is relatively constrained when compared to tertiary hospitals. Misinterpretation of the functional positioning of school hospitals may lead to low trust scores among university students toward these hospitals.

The accessible behavior score for university students was 14.55 ± 3.03, representing the lowest score among the dimensions analyzed. The primary obstacles to accessing school hospitals services may be the time conflict, as the opening hours of school hospitals usually coincide with students’ class hours. This may render school hospital services a secondary option for university students seeking care (15). Additionally, many school hospitals have yet to adopt appointment-based medical services, requiring students queue in-person, which imposes high time costs.

#### 3.2.3 Comparison of active use of health services in school hospitals

To investigate variation in active use of health service in school hospitals among university students, independent sample t-tests and one-way analysis of variance were used to compare scores by gender, age, education level, whether they are only child, family residence, household registration type, community type, medical habits, self-assessment of physical health, and tuition fees (Table 4).

**Table 4:** Comparison of scores for proactive use of health services by university students in school hospitals.

Characteristic	Category	Number of participants	Total score for proactive use of health services (mean ± standard deviation)	Statistic	*p*-value
Grade				*F* = 6.025	<0.01
	Freshman	286	62.4650 ± 10.00476		
	Sophomore	134	60.4179 ± 11.16540		
	Junior	165	57.4303 ± 11.78684		
	Senior	49	61.8980 ± 9.04444		
	Fifth-year undergraduate	15	60.9333 ± 9.66929		
Gender				t = 0.738	0.669
	Male	171	60.9883 ± 11.08416		
	Female	478	60.5753 ± 10.73375		
Age				*F* = 4.572	0.004
	≤18	70	62.2857 ± 10.34218		
	18–20	342	61.7749 ± 10.70145		
	20–22	200	58.6250 ± 11.08552		
	>22	37	58.7027 ± 9.71501		
Only Child				t = 2.400	0.017
	Yes	301	61.7874 ± 11.58712		
	No	348	59.7299 ± 10.02915		
Family residence				t = 0.668	0.504
	City where the school is located	98	61.3571 ± 11.31120		
	Other places	551	60.5644 ± 10.73664		
Household registration				*F* = 0.767	0.465
	Agriculture	347	60.5159 ± 10.11218		
	Non-agriculture (urban household registration)	300	60.9367 ± 11.60293		
	Others	2	52.0000 ± 7.07107		
Community type				*F* = 1.334	0.232
	High-end residential complexes/villas	21	62.3810 ± 12.10569		
	Ordinary residential housing estate	332	60.9699 ± 11.04258		
	Government/Enterprise/Institutional communities	37	63.0811 ± 9.81036		
	Affordable housing and other indemnificatory housing communities	9	55.8889 ± 11.83685		
	Unrenovated old urban communities	38	62.8158 ± 10.76495		
	Market town communities/township areas	56	60.5000 ± 9.16515		
	Rural areas	149	59.1745 ± 10.56494		
	Others	7	57.5714 ± 15.65095		
Medical treatment habits				*F* = 13.643	<0.01
	Have never been ill	96	63.6146 ± 10.63200		
	Have been ill but reluctant to seek medical treatment	136	56.7574 ± 9.11287		
	Have been ill and actively sought medical treatment	417	61.2902 ± 11.05492		
Self-assessment of physical health				*F* = 13.091	<0.01
	Very healthy	100	65.6000 ± 11.72927		
	Healthy	479	60.3612 ± 10.19070		
	Poor health status	68	56.2059 ± 10.97427		
	Very poor health status	2	44.5000 ± 13.43503		
University tuition fees				*F* = 4.597	0.001
	≤ 5,000	174	58.7586 ± 10.90470		
	5,001–10,000	457	61.6718 ± 10.60151		
	10,001–15,000	10	57.3000 ± 11.99120		
	15,001–20,000	7	49.4286 ± 10.34178		
	> 20,000	1	57		

Fifth-year undergraduates include thosein five-year programs such as medicine.

Table 4 reveals statistically significant differences in active use of school hospital health service behaviors by grade, age, only child status, medical habits, self-evaluation of physical health, and tuition fee. This indicates that these variables influence the behavior of university students’ active use of health services in school hospitals.

#### 3.2.4 Multiple linear regression analysis of the factors influencing active use of health services in school hospitals

This study further explores the primary factors influencing active use of health services in school hospitals using the total score of students’ active use as the dependent variable. The independent variables selected following univariate analysis include grade, age, only child status, healthcare-seeking habits, self-assessment of physical health, and tuition fees. The results are presented in Table 5.

**Table 5:** Factors influencing active use of health services in school hospitals among university students.

Variables	*B*	SE	*β*	*t*	*P*
Constant	74.996	3.029		24.758	<0.01
Self-assessment of physical health	−4.872	0.794	−0.234	−6.134	<0.01
Only child	−1.978	0.825	−0.091	−2.397	<0.05

*F* = 9.415; *p* < 0.001.

In conjunction with the results in Table 4, university students who rated themselves as very healthy had the best scores for active use of health services, while those who rated their health as very poor had the worst scores. This reflects a potential relationship between concerns regarding physical and mental health and the tendency to actively seek medical services (16) to maintain a healthy lifestyle. Specifically, university students who rated themselves as healthy are more likely to be proactive in managing their health. Their experiences with active use of health services, characterized by low-anxiety visits, are usually positive and relaxed (17), thereby strengthening their active engagement and gradually cultivating a positive cycle of “active use-satisfaction-reuse.”

Whether one is an only child also influenced active use of health services among university students, with only children scoring higher than their non-only child counterparts (Tables 4, 5). This discrepancy may be attributed to various factors influencing the medical-seeking behaviors of university students who are the only children, such as the tendency to seek medical attention promptly caused by higher family attention, and a family environment wherein perceptions of health issues are more pronounced than in non-only child families (18). This heightened awareness may lead only children to promptly seek medical attention for mild health concerns. Economic conditions may also influence the medical-seeking behaviors of only children, as better family finances might prompt them to actively address health issues (19).

## 4 Discussion

The research findings indicate that the level of active use of health services by university students in school hospitals is generally at general preference, and their scores regarding access to health services are weak.

### 4.1 Improving the accessibility of school hospital services

With the development of higher education in China and the continuous reinforcement of medical reforms, school hospitals have acquired new attributes. They have shifted from being traditional treatment centers for common ailments among students and staff to enhancing students’ overall health literacy. However, existing management regulations still face the issue of unclear functional positioning of school hospitals (14). In this context, clarifying the functional role of school hospitals is imperative. The content of basic services should be expanded to incorporate a dual-track service model of “basic medical care + health management,” aligned with student needs. In addition to routine diagnoses and treatments of cold, fever, and trauma, preliminary screenings for common mental health, vaccinations, and chronic disease follow-ups, are included in the health services of school hospitals. Hence, basic health problems can be solved on campus and reducing the students’ burden from seeking medical attention at large hospitals for minor illnesses. This study was approved by the Ethics Committee of Mudanjiang Medical University (No. 2025-MYKYYWF-09).

To optimize service hours at school hospitals, traditional administrative working hours should be reconsidered, orienting service provision around students’ schedules. Flexible service time mechanisms must be established and extending the reception time to evening to accommodate urgent health needs arising after school according to the characteristics of students’ concentrated courses during weekdays. Additionally, leveraging digital technology can pave the way for the development of a smart medical platform accessible via a school service application, facilitating online appointment bookings, inquiries, health consultations, and other functions to reduce waiting times, improve the efficiency of health services, and enhance the accessibility to health services provided by school hospitals.

### 4.2 Strengthening publicity and education of school hospital services

A strategy of classified publicity should be implemented based on the heterogeneous needs of student groups. For new student groups, information regarding the services offered by school hospitals and medical insurance reimbursement procedures should be included in the onboarding educational system, and basic cognitive construction should be completed through specialized lectures and physical distribution of educational materials. Guided by the research findings, targeted mental health education can be conducted for groups with poor self-rated health status, and assessing their actual health through offline exchange meetings to improve the pertinence and effectiveness of publicity content. Meanwhile, innovation in publicity channels and formats should be guided by university students’ acceptance habits. By leveraging platforms such as campus official accounts, video channels, and campus radio, institution can produce animated science popularization content and short videos to explain the functions of school hospitals and medical procedures. Effective use of publicity and educational materials can improve the health level of students seeking medical treatment (20). Organizing “school hospital open days” to ensure students are familiar with school hospitals through offline visits, equipment experience, and doctor-patient interaction, thereby enhancing their experience with medical services provided by school hospitals.

The present study found that many students preferred non-school hospitals as their first choice for treatment. Future efforts should also collect students’ feedback to gain an in-depth understanding of the reasons for the first choice of school hospitals. Establishing a regularized feedback mechanism can facilitate the collection of students’ perceptions regarding school hospital services through a combination of online surveys and offline symposiums This process can help delineate the critical factors that affect university students’ decision-making regarding active medical treatment, yielding a list of problems and improvement plans based on the survey results, enhance students’ participation, and trust in the improvement of school hospital services. Such enhancements can optimize students’ medical choices and the level of active use of health services in school hospitals.

### 4.3 Improving university students’ health initiative

Improving active use of health services in school hospitals by university students necessitates not only improvements in service provision and educational outreach but also efforts originating from the students themselves to shift from passive management to proactive health engagement, thereby enhancing their health behavior literacy. Students should devise personal health plans, including balanced diet, regular exercise, and proper sleep habits, formulating a personalized healthy eating based on the “Dietary Guidelines for Chinese Residents” to reduce dependence on fast foods. Furthermore, maintaining a healthy weight and selecting appropriate exercises based on personal interests will facilitate the integration of physical fitness into daily routines. It is essential to cultivate good sleep habits by going to bed and getting up early, avoiding late night overexertion (21).

Using smart mobile devices to record dietary intake, exercise, and sleep patterns can improve the sustainability of a healthy lifestyle. Students should proactively seek out diverse and practical health knowledge from authoritative channels, improving their ability to discerningly navigate the wealth of health information available in the era of big data. Additionally, acquiring essential self-identification methods and simple first-aid skills for common diseases is critical for managing emergencies. Regular participation in free health screenings organized by school hospitals is encouraged, examinations organized by the school hospital, enabling students to promptly amend their lifestyle choices according to the physical examination report and consult doctors to formulate corrective plans should issues like vision impairment or high blood pressure arise, thereby mitigating potential health risks.

## 5 Conclusion

The 16-item scale encompassing communication and dialogue, accessibility, risk assessment, and transparency, developed in this study based on the Delphi method demonstrates good reliability and validity, establishing a scientific and effective measurement tool for assessing university students’ the behavior toward active use of health service provided by school hospitals. Following an empirical investigation of 649 participants, recommendations include expanding and improving the basic service delivery of school hospitals, optimizing service operating hours, and creating smart medical platforms to enhance accessibility to health services. Additionally, strategies for classified publicity and innovative publicity channels and forms, alongside the organization and conduct of initiatives such as “school hospital open days,” should be implemented to strengthen student awareness of services provided by school hospitals. Furthermore, encouraging students to create personalize healthy diet, exercise, and sleep plans, alongside actively reserving practical health knowledge to enhance the factors influencing university students’ health initiative, thereby effectively improving their active use of health services.

## Data Availability

The original contributions presented in the study are included in the article/Supplementary material, further inquiries can be directed to the corresponding author/s.
