# Supplementary material for: Behavioral measures and factors influencing active use of health services in school hospitals by university students
Source: Front Public Health. 2025 Dec 12;13:1723284. doi: 10.3389/fpubh.2025.1723284 (PMC12741070; doi:10.3389/fpubh.2025.1723284)
Supplement: Supplementary file 1 [file Table_1.docx]

**大学生主动利用校医院卫生服务行为调查**

**调查问卷知情同意与匿名说明**

亲爱的同学：

您好！本问卷旨在开展大学生主动利用校医院卫生服务行为相关研究，所有数据仅用于学术分析，无任何商业或其他非研究用途。本问卷已通过牡丹江医科大学伦理审查委员会审查。

**知情同意说明**

1.您的参与完全基于自愿原则，可在问卷填写过程中任何阶段自由退出，无需说明理由，且不会对您造成任何不利影响。

2.问卷填写预计耗时10分钟，您可根据自身情况自主决定是否参与。

3.研究结果发表时，所有数据将以汇总形式呈现，不会单独识别任何个人信息。

**匿名与保密说明**

1.本问卷实行完全匿名制，问卷填写过程中不收集您的姓名、学号、联系方式等可识别个人身份的信息，所有回答仅以统计数据形式分析。

2.问卷数据将由研究团队统一加密存储，仅授权研究人员用于学术分析，严格遵守学术保密规范，绝不会向第三方泄露任何个人应答信息。

若您已阅读并理解以上说明，自愿参与本次调查，请点击“开始填写”；若不同意，可直接关闭页面，感谢您的关注与支持！

亲爱的同学，感谢你参与本次调查！本问卷主要分三部分:基本信息、背景信息和就医行为。你的回答有助于了解学生切实需求，以便创造一个更有帮助的学习环境，请选择最符合你的答案。谢谢！

**A 部分:基本信息**

1.专业: [单选题] *

| ○文科 |
| --- |
| ○理科 |
| ○工科 |
| ○医科 |
| ○农科 |
| ○商科 |
| ○其他 _________________ * |

2.年级: [单选题] *

| ○大一 |
| --- |
| ○大二 |
| ○大三 |
| ○大四 |
| ○大五（医学专业） |

3.性别: [单选题] *

| ○男 |
| --- |

○女

4.年龄 [填空题] *

_________________________________

**B 部分:背景信息背景信息主要包含你与家人的一些基本情况。你所填的信息将得到最严格意义上的保密。**

1.你的民族是 [单选题] *

| ○汉 |
| --- |
| ○蒙 |
| ○满 |
| ○回 |
| ○壮 |
| ○维 |
| ○藏 |
| ○其他 _________________ |

2.你是否是独生子女? [单选题] *

| ○是 |
| --- |
| ○否 |

3.如果非独生子女，请问你有几个兄弟姐妹（包括养父母的子女、继父母带来的子女，没有填0） [单选题] *

| ○兄 （ ）个 _________________ * |
| --- |
| ○弟 （ ）个 _________________ * |
| ○姐 （ ）个 _________________ * |
| ○妹 （ ）个 _________________ * |

依赖于（题目：你是否是独生子女?）第2个选项

4.你目前的政治面貌是 [单选题] *

| ○共青团员 |
| --- |
| ○群众 |
| ○民主党派 |
| ○共产党员（含预备党员） |

5.上大学前，你家居住在 [单选题] *

| ○本市(目前就读高校所在城市) |
| --- |
| ○外地: _________________ * |

6.上大学前，你家所在地属于 [单选题] *

| ○直辖市 |
| --- |
| ○省会城市 |
| ○地级城市 |
| ○县城 |
| ○镇区 |
| ○农村 |

7.上大学前，你的户口性质属于 [单选题] *

| ○农业 |  |
| --- | --- |

○非农业（城市户口）

○其他 _________________ *

8.上大学前，你家居住的社区类型是 [单选题] *

| ○高档商品房住宅小区／别墅 |
| --- |
| ○普通商品房小区 |
| ○经济适用房等保障性住房社区 |
| ○机关／企事业单位社区 |
| ○未经改造的老城社区 |
| ○集镇社区／镇区 |
| ○农村 |
| ○其他 _________________ * |

9.你的身高: （ ）厘米 [填空题] *

_________________________________

10.你的体重:（）公斤 [填空题] *

_________________________________

11.你的视力怎样? [单选题] *

| ○近视 |
| --- |

○一般

○较好

12.上大学以来，你是否有过生病就医的经历? [单选题] *

| ○没有生病过 |
| --- |

○生病过，但不想就就医

○生过病并积极就医

13．你通常会选择何种方式就医? [单选题] *

| ○自行购药 |
| --- |
| ○校医院 |
| ○当地三级以上医疗机构 |
| ○其他 _________________ * |

14.总体来说，你认为自己的身体状况如何? [单选题] *

| ○非常健康 |
| --- |
| ○健康 |
| ○健康状况较差 |
| ○健康状况很差 |

15.上一学年，你应缴纳的学费是 元／年 [单选题] *

| ○5000以下 |
| --- |
| ○5001-10000 |
| ○10001-15000 |
| ○15001-20000 |
| ○20000以上 |

16.上一学年，你的住宿费是 元／年 [单选题] *

| ○1000以下 |
| --- |
| ○1001-1500 |
| ○1501-2000 |
| ○2000以上 |

17.上一学年，你平均每月的生活费支出是_______元／月，其中，伙食费约____元，用于穿着打扮的费用约____元，用于就医的费用约____元，其他生活费约____元。 [填空题] *

18.上一学年，你的其他支出（不包括生活费）是______元／年，其中，用于补习培训班的费用约____元，用于各类考试的报名费用约____元，用于出国申请的费用约____元。 [填空题] *

19.上一学年经济来源项目:金额(元／年)（请填写具体金额，如无相应项目请填 0） [单选题] *

| ○父母支持（ ） 元／年 _________________ * |
| --- |
| ○奖学金（ ） 元／年 _________________ * |
| ○助学金 （ ） 元／年 _________________ * |
| ○助学贷款（ ）元／年 _________________ * |
| ○自己打工（ ）元／年 _________________ * |
| ○其他政府或学校补贴（ ）元／年 _________________ * |
| ○其他收入（ ）元／年 _________________ * |

**C 部分:校医院就医行为**

1.我可以和校医院的医护人员通过电话、线上APP等方式实现多渠道进行交流。 [单选题] *

| ○非常不同意 | ○不同意 | ○中立 | ○同意 | ○非常同意 |
| --- | --- | --- | --- | --- |

2.校医院医护人员会及时耐心地解答我的问题和疑虑。 [单选题] *

| ○非常不同意 | ○不同意 | ○中立 | ○同意 | ○非常同意 |
| --- | --- | --- | --- | --- |

3.校医院医护人员能认真倾听我的症状和需求。 [单选题] *

| ○非常不同意 | ○不同意 | ○中立 | ○同意 | ○非常同意 |
| --- | --- | --- | --- | --- |

4.我能够和医护人员共同决策最终治疗方案。 [单选题] *

| ○非常不同意 | ○不同意 | ○中立 | ○同意 | ○非常同意 |
| --- | --- | --- | --- | --- |

5.校医院是我的就医首选? [单选题] *

| ○非常不同意 | ○不同意 | ○中立 | ○同意 | ○非常同意 |
| --- | --- | --- | --- | --- |

6.寻求校医院服务在时间和地点上对我来说很方便。 [单选题] *

| ○非常不同意 | ○不同意 | ○中立 | ○同意 | ○非常同意 |
| --- | --- | --- | --- | --- |

7.我可以通过多种渠道（如校园宣讲、版面宣传、校园app）了解到学校医院服务。 [单选题] *

| ○非常不同意 | ○不同意 | ○中立 | ○同意 | ○非常同意 |
| --- | --- | --- | --- | --- |

8.当我需要就医时，寻求校医院造成的经济负担我完全可承受。 [单选题] *

| ○非常不同意 | ○不同意 | ○中立 | ○同意 | ○非常同意 |
| --- | --- | --- | --- | --- |

9.我认为校医院医生专业度很高。 [单选题] *

| ○非常不同意 | ○不同意 | ○中立 | ○同意 | ○非常同意 |
| --- | --- | --- | --- | --- |

10.我在校医院就医时能和校医生形成长期和谐的医患关系。 [单选题] *

| ○非常不同意 | ○不同意 | ○中立 | ○同意 | ○非常同意 |
| --- | --- | --- | --- | --- |

11.我认为在校医院就医的诊疗效果显著。 [单选题] *

| ○非常不同意 | ○不同意 | ○中立 | ○同意 | ○非常同意 |
| --- | --- | --- | --- | --- |

12.我认为校医院能够保护我的个人健康数据。 [单选题] *

| ○非常不同意 | ○不同意 | ○中立 | ○同意 | ○非常同意 |
| --- | --- | --- | --- | --- |

13.就医前我能获取清晰的校医院就医流程。 [单选题] *

| ○非常不同意 | ○不同意 | ○中立 | ○同意 | ○非常同意 |
| --- | --- | --- | --- | --- |

14.校医院收费标准公开透明。 [单选题] *

| ○非常不同意 | ○不同意 | ○中立 | ○同意 | ○非常同意 |
| --- | --- | --- | --- | --- |

15.校医生能够随时向学生解释疾病相关信息。 [单选题] *

| ○非常不同意 | ○不同意 | ○中立 | ○同意 | ○非常同意 |
| --- | --- | --- | --- | --- |

16.我可以随时查询自己在校医院的就医状态/记录。 [单选题] *

| ○非常不同意 | ○不同意 | ○中立 | ○同意 | ○非常同意 |
| --- | --- | --- | --- | --- |

**Investigation on College Students' Active Utilization of Health Service Behaviors in the University Hospital**

**Informed consent and anonymous explanation in the questionnaire survey.**

Dear student:

Hello! Informed consent and Anonymous Instructions for the Questionnaire Survey This questionnaire aims to conduct research on college students' active utilization of the health services provided by the university hospital. All data will only be used for academic analysis and have no commercial or other non-research purposes. This questionnaire has been reviewed and approved by the Ethics Review Committee of Mudanjiang Medical University.

**Informed Consent Statement**

1.Your participation is entirely voluntary. You can freely withdraw at any stage of the questionnaire filling process without giving a reason, and it will not cause you any adverse effects.

2.The questionnaire filling is expected to take 10 minutes. You can decide for yourself whether to participate based on your own situation.

3.When the research results are published, all data will be presented in an aggregated form and no personal information will be identified separately.

**Anonymity and Confidentiality Notice.**

1.This questionnaire is completely anonymous. During the filling process, no personal identifiable information such as your name, student number, or contact details will be collected. All responses will only be analyzed in the form of statistical data.

2. The questionnaire data will be uniformly encrypted and stored by the research team, and only authorized for academic analysis by researchers. We strictly adhere to academic confidentiality norms and will never disclose any personal response information to any third party.

If you have read and understood the above instructions and are willing to participate in this survey, please click "Start Filling". If you don't agree, you can directly close the page. Thank you for your attention and support!

Dear student, thank you for participating in this survey! This questionnaire is mainly divided into three parts: basic information, background information and medical treatment behavior. Your answer helps to understand the actual needs of students, so as to create a more helpful learning environment. Please choose the answer that best suits you. Thank you!

**Part A: Basic Information**

1. Major:[Single-choice question]*

| ○Liberal Arts |
| --- |
| ○Science |
| ○Engineering majors |
| ○Medical science |
| ○Agricultural Science |
| ○Business |
| ○Others _________________ * |

2. Grade: [Single-choice Question] *

| ○Freshman Year |
| --- |
| ○Sophomore Year |
| ○Junior Year |
| ○Senior Year |
| ○others _________________ * |

3. Gender: [Single-choice question] *

| ○Male |
| --- |

○Female

4. Age [Fill-in-the-blank question *

_________________________________

**Part B: Background Information Background information mainly includes some basic information about you and your family. The information you fill in will be kept confidential in the strictest sense.**

1. Your ethnicity is [single-choice question] *

| ○Han ethnicity |
| --- |
| ○Mongolian ethnic group |
| ○Manchu ethnic group |
| ○Hui ethnic group |
| ○Zhuang Ethnic Group |
| ○Uyghur |
| ○Tibetan |
| ○others _________________ |

2. Are you an only child? [Single-choice question] *

| ○Yes |
| --- |
| ○No |

3. If you are not an only child, how many siblings do you have (including the children of adoptive parents and stepparents, no 0 is filled in)? [Single-choice question] *

| ○Brother () _________________ * |
| --- |
| ○I () _________________ * |
| ○Sister () _________________ * |
| ○sister () _________________ * |

Rely on (Title: Are you an only child?) The second option

4. Your current political status is [single-choice question] *

| ○Member of the Communist Youth League |
| --- |
| ○Masses |
| ○Democratic parties |
| ○Communist Party members (including probationary members) |

5. Before going to university, your family lived in [single-choice question] *

| ○This city (the city where the current university is located) |
| --- |
| ○Out-of-town: _________________ * |

6. Before going to university, the area where your home is located belongs to [single-choice question] *

| ○Municipality directly under the Central Government |
| --- |
| ○Provincial capital city |
| ○Prefecture-level city |
| ○County town |
| ○Town area |
| ○Rural areas |

7. Before going to university, the nature of your household registration was [single-choice question] *

| ○Agriculture |  |
| --- | --- |

○Non-agricultural (urban household registration

○Others _________________ *

8. Before going to university, the type of community where your family lived was [single-choice question] *

| ○High-end commercial housing residential communities/villas |
| --- |
| ○Ordinary commercial housing community |
| ○Communities providing affordable housing and other types of subsidized housing |
| ○Community of government agencies/enterprises and public institutions |
| ○An unrenovated old town community |
| ○Market town community/town district |
| ○Rural areas |
| ○Others _________________ * |

9. Your height: () centimeters [fill-in-the-blank question *

_________________________________

10. Your weight: () kilograms [fill-in-the-blank question] *

_________________________________

11. How is your eyesight? [Single-choice question *

| ○Myopia |
| --- |

○General

○Better

12. Since you started college, have you ever had the experience of seeking medical treatment due to illness? [Single-choice question] *

| ○Have never been ill |
| --- |

○I was ill before, but I didn't want to seek medical treatment

○Have been ill and sought medical treatment actively

13．Which way do you usually choose to seek medical treatment? [Single-choice question *

| ○Buy medicine by oneself |
| --- |
| ○School Hospital |
| ○Local tertiary and above medical institutions |
| ○Others _________________ * |

14. Overall, what do you think of your physical condition? [Single-choice question*

| ○Very healthy |
| --- |
| ○Health |
| ○Poor health condition |
| ○My health condition is very poor |

15. The tuition fee you should pay for the last academic year was yuan per year. [Single-choice question] *

| ○Under 5000 |
| --- |
| ○5001-10000 |
| ○10001-15000 |
| ○15001-20000 |
| ○More than 20000 |

16. Last academic year, your accommodation fee was yuan per year. [Single-choice question] *

| ○Under 1000 |
| --- |
| ○1001-1500 |
| ○1501-2000 |
| ○More than 2000 |

17. Last academic year, your average monthly living expenses were _______ yuan per month. Among them, food expenses were approximately ____ yuan, expenses for clothing and grooming were approximately ____ yuan, medical expenses were approximately ____ yuan, and other living expenses were approximately ____ yuan. [Fill-in-the-blank question] *

18. Last academic year, your other expenses (excluding living expenses) were ______ yuan per year. Among them, the cost for tutorial classes was approximately ____ yuan, the registration fee for various examinations was approximately ____ yuan, and the cost for applying to study abroad was approximately ____ yuan. [Fill-in-the-blank question] *

19. Source of income for the previous academic year: Amount (yuan/year) (Please fill in the specific amount. If there is no corresponding item, please fill in 0) [Single-choice question] *

| ○Parental support () yuan/year _________________ * |
| --- |
| ○Scholarship () yuan per year _________________ * |
| ○Grant () Yuan/year _________________ * |
| ○Student loan () yuan/year _________________ * |
| ○Self-employment () yuan/year _________________ * |
| ○Other government or school subsidies () yuan/year _________________ * |
| ○Other income () yuan/year _________________ * |

**Part C: Medical treatment behavior at the university hospital**

1. I can communicate with the medical staff of the school hospital through multiple channels such as phone calls and online apps. [Single-choice question] *

| ○Strongly disagree | ○Disagree | ○Neutral | ○agree | ○totally agree |
| --- | --- | --- | --- | --- |

2. The medical staff of the school hospital will answer my questions and doubts in a timely and patient manner. [Single-choice question *

| ○Strongly disagree | ○Disagree | ○Neutral | ○agree | ○totally agree |
| --- | --- | --- | --- | --- |

3. The medical staff at the school hospital listened carefully to my symptoms and needs. [Single-choice question *

| ○Strongly disagree | ○Disagree | ○Neutral | ○agree | ○totally agree |
| --- | --- | --- | --- | --- |

4. I can make the final treatment plan together with the medical staff. [Single-choice question] *

| ○Strongly disagree | ○Disagree | ○Neutral | ○agree | ○totally agree |
| --- | --- | --- | --- | --- |

5. Is the school hospital my first choice for medical treatment? [Single-choice question] *

| ○Strongly disagree | ○Disagree | ○Neutral | ○agree | ○totally agree |
| --- | --- | --- | --- | --- |

6. Seeking services from the school hospital is very convenient for me in terms of time and place. [Single-choice question] *

| ○Strongly disagree | ○Disagree | ○Neutral | ○agree | ○totally agree |
| --- | --- | --- | --- | --- |

7. I can learn about the services of the school hospital through various channels, such as campus lectures, board promotions, and campus apps. [Single-choice question] *

| ○Strongly disagree | ○Disagree | ○Neutral | ○agree | ○totally agree |
| --- | --- | --- | --- | --- |

8. When I need medical treatment, I can fully afford the financial burden caused by seeking medical care at the school hospital. [Single-choice question *

| ○Strongly disagree | ○Disagree | ○Neutral | ○agree | ○totally agree |
| --- | --- | --- | --- | --- |

9. I think the doctors in the school hospital are highly professional. [Single-choice question] *

| ○Strongly disagree | ○Disagree | ○Neutral | ○agree | ○totally agree |
| --- | --- | --- | --- | --- |

10. When I seek medical treatment at the school hospital, I can form a long-term and harmonious doctor-patient relationship with the school doctors. [Single-choice question] *

| ○Strongly disagree | ○Disagree | ○Neutral | ○agree | ○totally agree |
| --- | --- | --- | --- | --- |

11. I think the medical treatment effect at the school hospital is remarkable. [Single-choice question] *

| ○Strongly disagree | ○Disagree | ○Neutral | ○agree | ○totally agree |
| --- | --- | --- | --- | --- |

12. I think the school hospital can protect my personal health data. [Single-choice question] *

| ○Strongly disagree | ○Disagree | ○Neutral | ○agree | ○totally agree |
| --- | --- | --- | --- | --- |

13. Before seeking medical treatment, I can obtain a clear medical process at the school hospital. [Single-choice question] *

| ○Strongly disagree | ○Disagree | ○Neutral | ○agree | ○totally agree |
| --- | --- | --- | --- | --- |

14. The charging standards of the university hospital are open and transparent. [Single-choice question] *

| ○Strongly disagree | ○Disagree | ○Neutral | ○agree | ○totally agree |
| --- | --- | --- | --- | --- |

15. The school doctor can explain disease-related information to students at any time. [Single-choice question] *

| ○Strongly disagree | ○Disagree | ○Neutral | ○agree | ○totally agree |
| --- | --- | --- | --- | --- |

16. I can check my medical treatment status/records at the school hospital at any time. [Single-choice question] *

| ○Strongly disagree | ○Disagree | ○Neutral | ○agree | ○totally agree |
| --- | --- | --- | --- | --- |
